# Process Waters from Hydrothermal Carbonization of Sludge: Characteristics and Possible Valorization Pathways

**DOI:** 10.3390/ijerph17186618

**Published:** 2020-09-11

**Authors:** Michela Langone, Daniele Basso

**Affiliations:** 1 Laboratory of Technologies for the efficient use and management of water and wastewater, Italian National Agency for New Technologies, Energy and Sustainable Economic Development (ENEA), 00123 Roma, Italy; 2 HBI S.r.l., 39100 Bolzano, Italy; d.basso@hbigroup.it

**Keywords:** digestate, hydrothermal carbonization (HTC), HTC chemicals, process waters, sewage sludge

## Abstract

Hydrothermal carbonization (HTC) is an innovative process capable of converting wet biodegradable residues into value-added materials, such as hydrochar. HTC has been studied for decades, however, a lack of detailed information on the production and composition of the process water has been highlighted by several authors. In this paper the state of the art of the knowledge on this by-product is analyzed, with attention to HTC applied to municipal and agro-industrial anaerobic digestion digestate. The chemical and physical characteristics of the process water obtained at different HTC conditions are compared along with pH, color, organic matter, nutrients, heavy metals and toxic compounds. The possibility of recovering nutrients and other valorization pathways is analyzed and technical feasibility constraints are reported. Finally, the paper describes the main companies which are investing actively in proposing HTC technology towards improving an effective process water valorization.

## 1. Introduction

**Figure 1:**
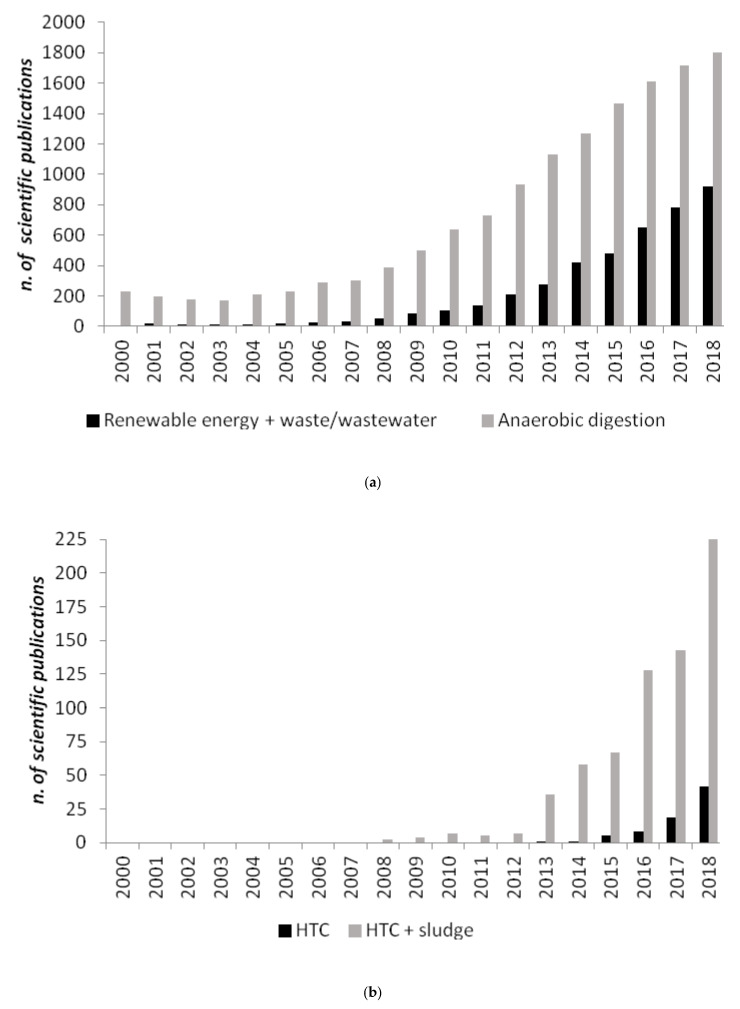
Number of scientific publications containing as keywords: (**a**) “renewable energy and waste/wastewater” and “anaerobic digestion”; (**b**) “hydrothermal carbonization (HTC)” and “hydrothermal carbonization (HTC) and sludge”, based on the NBCI bibliographic database from 2000 to 2018 [1].

Research in renewable energy production from bioresources, such as organic resources and waste, is constantly increasing in intensity and quality, pushed by the current rate of exploitation of fossil fuels and their related impacts and green house gases (GHGs) emissions on the environment. The use of anaerobic digestion (AD) for the energetic utilization of agro-industrial residues and even biodegradable municipal wastes is raising year after year (Figure 1a). The main products of the AD are biogas and digestate. While biogas is a valuable source of energy, as it can be used in combined heat and power (CHP) generators or upgraded to biomethane for vehicles fuel and immission into the natural gas grid, after undergoing an upgrading process, digestate in some contexts could represent a problem to the environment or can sometimes encounter disposal-related issues.

Digestate is a heterogeneous solid–liquid by-product produced in large amounts as result of the AD process [2]. It contains a high proportion of mineral nitrogen (N), mainly in the form of ammonium, together with other macro- and micro-elements necessary for plant growth, which make it exploitable as an organic fertilizer. Furthermore, its organic residual content makes it suitable as organic amendment, improving the physical, chemical and biological properties of the soil [3]. According to the current European legislation, when AD is applied to pre-selected biowastes, digestate can be sent for direct soil application, reducing the needs of chemically produced fertilizers [4]. Often, various treatment options can be applied both to optimize transport and application conditions, such as solid/liquid separation, composting, drying, thermal concentration, physical–chemical treatment [5] and pelleting [6], as well as to treat it in order to reduce the nitrogen content [7]. However, the amount of digestate that can be applied to the field is limited according to its nutrient content, its quality and, in some countries, according to the lack of fertile lands [4]. All these variables represent limiting factors of the application of digestate as organic fertilizer to soils. Moreover, depending on the size of the biogas plant, on the transportation costs and on the type of initial feedstocks, application of digestate on the field is not always economically feasible or allowed. In this sense, the importance of digestate management has been recognized by research and policy institutions, requiring a more sustainable approach to be adopted, mainly considering this waste as a feedstock for further bio-transformation [8] and thermal transformation [9] in a circular economy framework.

Recently, a relatively new technology, hydrothermal carbonization (HTC), has been proposed as a novel solution to treat biomass residuals, and among those digestates [10] producing a solid value-added product is hydrochar, which can be used as a high-quality alternative fuel or as a soil amender [11] and carbon sink [12]. Even if HTC has been known for about a century, only over the past two decades has it received greater attention in biomass conversion research (Figure 1b). A wide range of biomass feedstocks, including cellulose [13], bamboo [14], loblolly pine [15], fruit waste [16], olive waste [17], tomato peel [18], agro-industrial waste [19], walnut shell and sunflower stem [20], microalgae [21,22], municipal solid food waste [23,24], distiller’s grains [25], animal manure [26,27], have been applied in HTC to gain fuels or materials. Interestingly, HTC has also been applied to municipal sewage sludge [28,29,30,31], anaerobically digested sewage sludge (ADSS) [32] and anaerobically digested biomasses [10].

HTC has attracted a great deal of interest primarily because it uses water as a reaction medium, which is a non-toxic, environmentally benign, and inexpensive reactant. This advantage is further increased when treating wet biomass, such as sludge and digestate, where water is inherently present. The benefits gained by carbonizing the digestate by HTC has been investigated by several research groups [33,34]. The integration of biogas and biochar promises several synergies, improving energy recovery from biomass wastes. However, large quantities of process waters are obtained as a by-product of the HTC process that have to be managed, treated and possibly valued [35].

**Figure 2:**
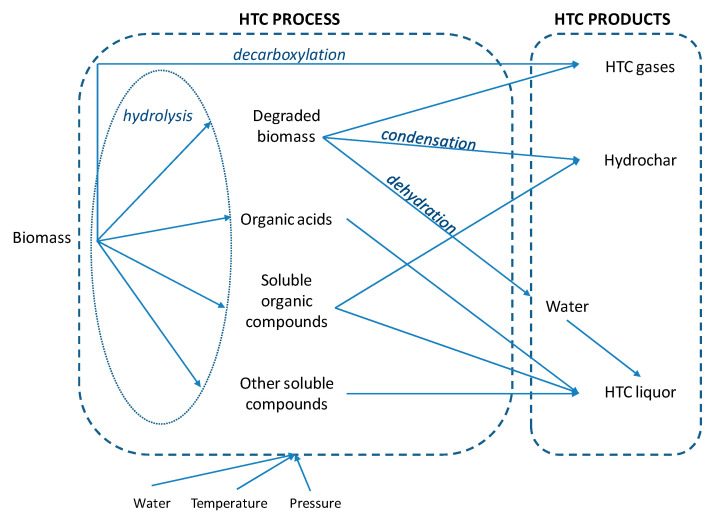
HTC process.

HTC is a wet thermochemical process which involves the application of heat and pressure to convert raw material in the presence of water into a carbonaceous biofuel. A series of hydrolysis, condensation, decarboxylation and dehydration reactions occur during HTC (Figure 2). Reaction temperatures are applied within a range of 180–250 °C and pressures are maintained above the corresponding saturation pressure (10–50 bar) to ensure the liquid state of water [36]. Treatment times were reported to vary between a few minutes to several hours by many authors. In comparison to pyrolysis, HTC requires wet feedstock and therefore the digestate, which usually has a high moisture content of 54.3–98.5% [3], does not need to be dried prior to or during the process, saving a substantial amount of energy. Thus, the water contained in the raw digestate can be directly used as a reaction medium [37].

However, many studies have largely focused on the properties of the resulting solid product (hydrochar), energy recovery, hydrochar yields and its combustion characteristic. On the contrary, very few studies began to characterize the HTC process waters, oriented towards their valorization, changing them from wastes into products with a much greater value (chemicals, fuels, energy, as well as many other products beneficial for a local economy). This gap was highlighted by Owsianiak et al. [38] in their life cycle assessment (LCA) analysis, where it is shown that there are still considerable uncertainties about both the composition of HTC process waters and their potential valorization, recommending that technology developers measure the composition of process waters, especially with respect to possible application as fertilizer or for chemicals recovery. Thus, this manuscript presents the current state of the art for hydrothermal technologies in the digestate processing field for pretreatment and conversion of biomass and sewage sludge to chemical or fuel precursor, aiming to underline the possibility of valorizing the liquid by-product.

## 2. Digestate

Digestate is characterized by a high humidity content, commonly higher than 90%. The whole digestate composition depends on the type of input materials loaded into the biogas plant, as well as on the type of AD reactor [39]. Various types of feedstock have been reported in the literature, in particular, municipal primary sludge and waste-activated sludge [40], cattle manure [41], pig manure [42], solid organic waste [43], agricultural residues [44] and combinations of feedstocks, such as agricultural waste and dairy cow manure [45], sewage sludge and the organic fraction of municipal solid wastes [46], dairy cattle slurry and industrial meat-processing by-products [47], chicken manure and corn stover [48], etc. Table 1 contains indicative and non-exhaustive examples of the variability of digestate with respect to the initial feedstocks. 

Municipal ADSS are characterized by a lower percentage of biodegradable carbon content as compared with digestate from other biomasses, which can be explained by the previous conversion of organic compounds into CO_2_ in the biological processes occurring during the wastewater treatment line. Digested sludge from an agricultural biogas plant has a higher carbon content due to the characteristics of the raw biomass used as feedstock. Nitrogen concentrations in pig slurry co-digestion plants are quite similar, whereas in biogas plants which treat organic wastes, the nitrogen concentrations vary strongly. Lower nitrogen contents are present in digestate from cow manure co-digestion plants and municipal biogas plants. The reason for this is mostly the different nitrogen concentrations in the corresponding substrates. Furthermore, the process design, e.g., the amount of fresh water and recirculation effluent in use, may influence the total nitrogen concentrations.

The analysis of the results showed that, independently from the initial feedstock, digestate has a relatively high metals content, in particular Fe, Al and Ca, which could interfere with phosphorous recovery; this represents one interesting and viable possibility to valorize this feedstock through HTC. Iron concentrations in municipal sewage sludge are high because, for example, it is added in sewage pumping stations to control odors and corrosion, and further iron or aluminum salts are added in municipal wastewater treatment to remove phosphate from wastewater with the intention to prevent eutrophication of the surface [49]. Instead, in agro-industrial biogas plants the use of micronutrients is being consolidated (i.e., Mo, Ni, Co, Se, Fe) to improve biogas production [50].

In order to optimize its management, raw digestate commonly undergoes to mechanical solid–liquid separation by means of a screw press or a decanter centrifuge, where a fiber/solid fraction and a liquid fraction are separated. The solid fraction of digestate usually displays significantly higher concentrations of phosphorus, organic nitrogen, organic carbon, whereas liquid fractions tends to have higher concentrations of available nitrogen and orthophosphate. Typical ranges for the distribution of the main constituents between the fiber/solid and the liquor are provided by several studies [51,52]. It is worth noting that the HTC process can be applied to the whole digestate or to the dewatered solid digestate.

## 3. Hydrothermal Carbonization

**Figure 3:**
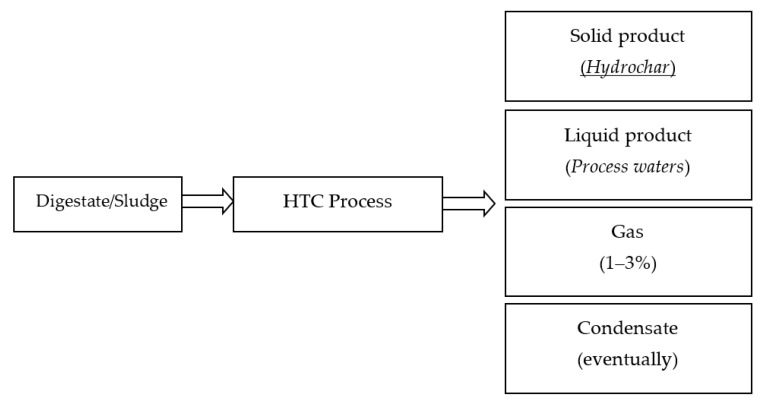
HTC by-products.

HTC is a valid strategy for treatment and valorization of biomass residuals, especially those with a high water content (up to 80%) such as digestate. The HTC process mainly results in three types of products: a solid hydrochar and aqueous and gaseous by-products (Figure 3).

During HTC organic substrates in sealed containers are mixed with water under mild temperature conditions (180–250 °C) and pressure slightly higher than the water saturation pressure to ensure that water remains in its liquid state. Under this range of temperature, the water is in subcritical conditions and acts as an effective solvent, catalyst and reactant for hydrolytic conversion and extractions [57] from wet biomasses and waste streams. Typical chemical reactions occurring during HTC are hydrolysis, dehydration, decarboxylation, aromatization, condensation and polymerization [11,58]. High moisture content favored decarboxylation and hydrolysis reactions [59]. Mainly, the carbon content of the feedstock increases, achieving a higher calorific value [60] and producing a solid value-added product, namely hydrochar. 

Hydrochar can be defined as a homogenized, carbon-rich and energy-rich solid fuel, biologically sterilized due to thermal treatment, with a similar energy density as lignite, which contains most of the organic compounds originally present within the feedstock. Its characterization, production yield and higher heating value (HHV) depend on feedstock type and HTC process parameters [11,61]. Its utilization as a stable energy-rich solid fuel in combustion processes is widely recognized [28]. However, in recent years, other uses of the hydrochar have emerged: it can be exploited as a soil amendment and nutrient source, as low-cost adsorbent for contaminants in aqueous solutions, for the generation of nano-structured materials [62], as precursor for activated carbons [63], as a catalyst [64], as well as for CO_2_ sorption and sequestration [65]. Moreover, hydrochar has been applied in the AD process to promote the biomethane production, by acting both as support for bacteria colonies, conductor for electron transfer among species, sorbent for indirect inhibitors, and reactant in chars labile carbon methanization [66,67]. Further, its pelletization has been studied by Wang et al. [24].

HTC treatment also produces a process water, as a result of the amount of water initially present within the HTC reactor. Process waters are a liquor rich in dissolved organic components and inorganic salts [58]. Like the solid product, process waters are highly dependent on feedstock type and HTC process parameters [35]. They can be recycled to the HTC process or further treated. However, in the contest of maximizing energy and resource recovery, HTC process waters need to be valorized. Finally, HTC also produces a very small amount of gas, about 1–3% of the mass of the raw material, which is mainly composed of CO_2_ with traces of CO [23]. Depending on HTC process conditions, oily molecules (water-insoluble) may also be generated in hydrothermal processes [68,69]. Furthermore, on the industrial scale, when steam is used as a heating medium, a condensate rich in volatile organic compounds can be obtained from HTC [70].

The formation, the yields and the composition of solid, liquid and gaseous by-products depend on the biomass being processed, as well as on the processing HTC conditions, such as residence time and temperature [34] and biomass-to-water ratio (b/w) [59].

Usually, reaction time can vary from a few minutes up to several hours (12–20 h), even if most of the reactions seem to last within the first reaction hour [71]. However, it has been observed that both time and temperature influence product characteristics [61], the latter being the most influencing process parameter [58].

Further, the addition of some additives, such as acids, bases and salts, can affect the characteristics of products formed, having an effect on the degree of carbonization (described by the H/C ratio), the HHV and the extraction of certain elements. Moreover, additives may act as catalysts, thus reducing the HTC reaction temperatures and pressures [72,73]. The water pH has a significant impact on the hydrochar yield, its HHV and ash content [73], and, as a consequence, the addition of selected salts can increase the HHV of the obtained hydrochar [74]. The most frequently reported mineral acids are hydrochloric and sulfuric acid [72,73], while the organic acids used are acetic and citric acid [73,75,76]. Stemann et al. [77] showed that by process water recirculation, organic acids in the liquid phase catalyze HTC reactions. NaOH, Ca(OH)_2_ are used as bases [72,78], while NaCl and CaCl_2_ [72] and Ca propionate, Ca acetate, Mg acetate, Ca lactate, Li chloride, Ca chloride and Ca formate are commonly used as salts [74]. CaO additive has been employed in near-critical water to act as CO_2_ absorber via carbonation [59], while zeolite has been used as a catalyst in the HTC process, increasing the energy and carbon recovery in hydrochar from digestate [79].

## 4. Hydrothermal Carbonization of Digestate

HTC has been applied for a variety of feedstocks, including digestate produced by means of AD of different feedstocks (Table 2). Digestate can be treated directly in the HTC process as wet feedstock, or it can be treated after a solid/liquid separation process. In some studies, despite the advantage of HTC in treating wet feedstock, the digestate was dried in an oven for two–three days at 60 °C to ensure a homogenous dry matter content and consistent carbon content [80]. 

Examples of HTC applications will be considered here to illustrate the increasing importance of the processes in sludge and digestate treatment.

As far as municipal anaerobically digested sewage sludge (ADSS) is concerned, both the HTC solid and liquid by-products have been characterized, following the fate of carbon [23], nitrogen and phosphorus [33,81,96] in the product streams. The speciation of phosphorus in sludge and hydrochar has been thoroughly studied [76,94]. Kim et al. [32] showed that HTC can be used to successfully treat ADSS, producing a carbonaceous solid product, hydrochar, with a lower moisture content and HHV than the raw digestate, making it a viable alternative to fossil solid fuels. The fuel characteristics and combustion behavior of this type of hydrochar were both studied by He et al. [28], while its adsorption behavior was investigated by Alatalo et al. [87]. Aragón-briceño et al. [34] studied the valorization of the HTC process waters obtained from an ADSS, showing that HTC can increase the biomethane potential production of the whole HTC by-product (hydrochar + processed water) and in process waters alone. Wirth et al. [89] studied the influence of AD temperature and organic loading rate on the continuous anaerobic treatment of process waters from HTC of ADSS. Valorization of HTC process waters from ADSS through AD was further evaluated by Yu et al. [90]. HTC condensate from steam-derived HTC of ADSS was further characterized and valorized by AD in the research conducted by Wirth and Reza [70]. The improvement of digestate dewaterability after an HTC process has also been studied [32,82]. Kim et al. [32] showed that the HTC process breaks up the physical structure of the sludge, converting the bound water to free water within the sludge. The use of catalysts for the HTC process was investigated by Escala et al. [82], who used citric acid obtaining a hydrochar with a slightly higher C content compared to the samples without a catalyst and a lower HTC reaction time. The effect of calcium oxide on the evolution profile and characteristics of gas, solid and liquid by-products of HTC from ADSS was reported by He et al. [59]. The phosphorous and heavy metal distribution in the by-products of dewatered ADSS was investigated by several authors [83,84]. In order to reduce the considerable emissions of N-containing pollutants from combustion of the sewage sludge-derived solid fuel, an integrated system of hydrothermal deamination and air stripping from HTC water processing was developed to effectively remove and recover nitrogen from dewatered sewage sludge [59]. Similarly, Shen et al. [84] proposed a combined HTC and CO_2_ gasification process that could be coupled with a struvite precipitation or air-stripping treatment of HTC process waters in order to recover nitrogen. Recently, Yu et al. [81] proposed HTC pretreatment of ADSS coupled with MgNH_4_PO_3_ · 6 H_2_O (MAP) crystallization for the simultaneous recovery of phosphorus and nitrogen.

With reference to the HTC of digestate from agro-industrial waste, some experiences are reported in the literature. Funke et al. [33] studied a combined system that combines AD of wheat straw with HTC of digestate, doubling the energy recovery obtained by the single AD process. Indeed, about 50% of the AD total energy input can be recovered in the hydrochar, which represents a valuable fuel with an estimated HHV (on a dry matter basis) of 31.5 MJ kg^−1^. Similarly, Oliveira et al. [26] applied HTC to digestate of agricultural residues, producing a solid fuel (hydrochar) comparable to brown coal, which could be used to complement farm-based biogas plants [26]. Mumme et al. [10] applied HTC at an anaerobically thermophilic digested maize silage, showing that relatively mild conditions (190 °C, 2 h) are suitable for producing hydrochar which is potentially interesting as an alternative fuel or soil conditioner. Interestingly, Mumme et al. [79] studied the catalytic effects of zeolite on carbonization of agro-industrial digestate, producing a hydrochar−zeolite composite with potential applications ranging from energetic use to soil amendment and additive in bioprocesses (e.g., growing media). The use of hydrochar obtained from anaerobically thermophilic wheat straw digestate as adsorbent in AD processes was studied by Mumme et al. [66], who showed that the addition of hydrochar increased the methane yield by 32% due to the ability to catalyze AD by mitigation of mild ammonia inhibition, support of growth of archea and methanization of the hydrochar’s labile carbon. 

HTC process water composition from an agro-industrial digestate was analyzed by Ekpo et al. [91]. Becker et al. [80] applied the HTC to wheat straw digestate and studied the composition of process waters, in terms of major organic components. Oliveira et al. [26] proposed that the process waters can be recycled in the biogas plant, reducing their environmental impacts and increasing the energy efficiency of the whole process. The combined system, with AD as primary treatment and HTC as post-treatment applied to the solid digestate, has also been investigated to treat microalgae by Nuchdang et al. [93], proving that applying HTC to digestate and recycling the HTC process water to the primary AD would increase methane production by 40%. The possibility to valorize the HTC process water through AD has also been considered for HTC liquor produced from anaerobically digested corn silage [92]. 

Recently, digestate from organic fraction of municipal solid waste (OFMSW) was treated by HTC by Reza et al. [94], obtaining an energy–dense solid hydrochar and a process water with mainly organic acids and amino acids. Zhou et al. [97] underlined in their study the synergistic effect of the combination of AD and HTC, in order to evaluate a sustainable treatment for food waste in China.

Life cycle assessment (LCA) analyses of HTC technology have been carried out in order to identify the processes configuration with the largest potential, and to drive the environmentally conscious design of future HTC. Berge et al. [23] showed how the impacts of HTC of food waste and combustion of the resulting hydrochar in a power plant depend mainly on process waters treatment and valorization and the type of energy that is substituted. Similarly, Owsianiak et al. [38] using LCA, showed that HTC of green waste, food waste, organic fraction of municipal solid waste (MSW), and digestate followed by energy recovery, when hydrochar is used as solid fuel, is a potential technology to treat biowaste, underlining that both process waters and hydrochar compositions are important parameters influencing environmental performance. For example, across the life cycle impact, emissions of metallic elements from HTC process water discharge drove toxic impacts on human health and ecosystems, whereas the use of hydrochar as fuel, substituting energy derived from fossil sources, such as anthracite or lignite, resulted in the best environmental performance.

## 5. Hydrothermal Carbonization Process Water Characterization

The yields and the composition of the HTC process waters depend on the biomass being processed as well as on the HTC processing conditions, mainly temperature, reaction time, and ratio of dry biomass to water (b/w). The yield of process waters was found to be in the range of 25–55 wt.% by He et al. [59], who performed HTC on dewatered anaerobically digested sewage sludge using HTC temperature higher than 200 °C. The yield tends to increase when increasing both reaction temperature and the initial moisture content. This large range of values was attributed to the dissolution and decomposition of organic matter during the HTC process. Liquid yields of 17% and 30% were reported by Ekpo et al. [91], when treating agro-industrial digestate at 170 and 250 °C, respectively, as results of a combination of solubilization of inorganics and an increase in the production of soluble organic hydrocarbons. 

The management of HTC process waters needs to be evaluated according to its quality. In general, HTC process waters contain high concentrations of organic matter as indicated by TOC (Total Organic Carbon), soluble COD (Chemical Oxygen Demand) and BOD_5_ (Biochemical Oxygen Demand) values, and the relative abundance of nutrients (N, P, K) [35]. Table 3 summarizes the main findings reported in the literature for HTC process waters from municipal sewage sludge digestate and agro-industrial digestate, respectively. 

### 5.1. pH and Color

The pH of HTC process waters from biomass is usually around 4.5 or below [35], except when treating ADSS, for which the pH is generally alkaline [23]. This is mainly due to the high buffering capacity of the digestate. However, lower pH values of process waters obtained from dewatered ADSS were reported by Yu et al. [81], in the range of 5.6–6.8. HTC process waters from MSW digestate has a pH of around 8.0, while pH values in the range of acidity have been reported for process waters obtained from digestate of corn silage [92] and wheat straw [80], probably due to a lower buffering capacity of initial biomass. Ekpo et al. [91] reported pH values of HTC process waters from agro-industrial digestate close to 8.0, due to the presence of manure as feedstock of a biogas plant, which gives a higher buffering capacity. Furthermore, in process waters obtained from HTC of ADSS, Aragón-briceño et al. [34] showed that pH can be influenced by the HTC temperature, due to the presence of volatile fatty acids (VFAs), amino acids and ammonia nitrogen which are generated during the HTC treatment. The authors reported higher pH values at lower HTC temperatures (pH of 9.15 at 160 °C vs. 8.08 at 250 °C). 

Process liquids from HTC are dark in color [94], a yellowish to dark brownish liquid [59]. Recently, Xu and Jiang [69], treating urban sewage sludge, reported that the color can be used as a crucial index to evaluate the purification of the process water, as it can be associated with the dissolution and decomposition of organic matter during HTC. It becomes lighter from black-brown to yellowish-brown with increasing temperature from 180 to 300 °C, respectively, while the content of organic matter decreases. Similar results were reported by He et al. [59] for dewatered ADSS. 

### 5.2. Organic Compounds

Several organic compounds have been detected in the HTC process waters as a result of thermal degradation of the feedstock [23]. Table 4 summarizes compounds identified in the HTC process water from HTC of ADSS and gives an overview of their possible main applications in the chemical industry.

HTC process waters from municipal ADSS could contain up to 30% of the initial carbon (C) present in the feedstock [23]. A carbon fraction of 17% has been reported in the HTC process waters from agro-industrial digestate [26]. However, the C content distribution depends on both the initial characteristics of sludge and on the HTC process conditions. Indeed, using an anaerobic granular sludge (AGS), Yu et al. [90] reported a higher C content in the HTC process waters, from 37% at 160 °C to 62% at 240 °C, due to the high (volatile solid) content in the AGS as compared with a suspended sludge.

Both TOC and soluble COD (SCOD) concentrations in HTC process waters from ADSS can vary in a wide range from 4000 mg L^−1^ [23] to 24,000 mg L^−1^ [34], and from 10,000 mg L^−1^ [23] to 64,000 mg L^−1^ [59], respectively. HTC process temperature plays an important role in determining C content in process waters. Aragón-briceño et al. [34] reported that the concentration of SCOD in the HTC process waters from ADSS increased 7-fold, increasing from 1843 in the raw digestate to 12,642 mg L^−1^ after 160 °C treatment, to 12,992 mg L^−1^ after 220 °C treatment, and decreasing to 12,164 mg L^−1^ after 250 °C treatment. A similar trend was observed for TOC that increased 10-fold after HTC treatment, from 461 to 4879 mg L^−1^. Similarly, for temperatures below 200 °C, treating a mixture of primary and secondary sludge, Qiao et al. [98] reported that SCOD of process water increased with increasing temperature, from 120 to 190 °C. However, after more than 30 min of heating, there was only a small increase in SCOD concentration. Up 240 °C, Yu et al. [81] reported a steady increase of TOC with the increase in the process temperature treating an ADSS. For temperatures higher than 200 °C, in their study, He et al. [59] showed that TOC and COD of process waters from ADSS decreased from 24.07 and 63.9 g L^−1^ to 12.51 and 30.4 g L^−1^, respectively, by increasing the temperature from 200 to 380 °C, while inorganic carbon (IC) was accumulated from 0.48 to 3.67 g L^−1^. This suggests that for temperature higher than 200 °C, the gasification process became faster than hydrolysis and dissolution of organic substances, while carbon underwent gradual mineralization.

Citric acid can be used as catalyst for the reactions in the HTC treatment, with the effect of increasing the COD content in process waters [82]. Furthermore, CaO, employed as additive for CO_2_ absorber via carbonation in near-critical water, has been reported to affect process waters composition [59]. CaO created alkaline hydrothermal conditions which promoted solubility of organic compounds, increasing TOC and COD concentrations in process waters from 18,630 up to 55,800 mgL^−1^, respectively. However, since the gasification process was also increased, TOC and COD dropped to 16,610 and 49,400 g/L, respectively.

HTC process waters from digested agricultural residues are characterized by high values of TOC concentrations. Oliveira et al. [26], analyzing process liquors from dewatered agro-industrial digestate and a combination of it with other biomasses (e.g., corn silage, poultry manure, a mixture of straw and manure, dry straw, cabbage and dough), reported TOC concentrations in the range of 13–26 g L^−1^. Similarly, wheat straw digestate leads to a TOC concentration between 5500 and 9500 mg L^−1^ after HTC under the applied conditions (190–270 °C, 6 h) [80]. Similarly to ADSS, wheat straw digestate displayed a significant increase of the TOC concentration between 190–230 °C, while a decreasing tendency was observed between 250 and 270 °C [80].

The concentration of volatile fatty acids (VFAs) in process waters also increased with temperature. Aragón-briceño et al. [34], in their study, reported VFA concentrations of 191, 406 and 715 mg L^−1^ for 160 °C, 220 °C and 250 °C treatments, respectively. Acetic acid, present as a product of hydrolysis, was the main constituent of the VFAs produced in all the treatments. The same has been reported by Wirth et al. [89]. They observed that the dominating organic compound was acetic acid with a concentration of 2.06 g L^−1^, accounting for 6.2% of TOC of process waters. Propionic acid and butyric acid showed concentrations of 0.12 g L^−1^ and 0.03 g L^−1^, respectively. Similar results have been reported by Yu et al. [90]. Higher percentages of VFAs have been measured in the HTC process waters obtained treating digested agricultural biomasses. Treating wheat straw digestate at several HTC conditions (190–270 °C, 6 h), acetic acid was reported to remain largely constant over the process, ranging between 1000 mgL^−1^ (190 and 230 °C) and 1200 mgL^−1^ (250 and 270 °C). Propionic acid demonstrates a significant increase from 70 to 130 mgL^−1^ with increasing process temperatures [80]. The presence of acetic and propionic acids was further reported in the process liquor obtained from HTC (220 °C, 6 h) of corn silage digestate, in concentration of 5260 and 340 mgL^−1^, respectively.

Finally, HTC causes the release of proteins and carbohydrates in the process waters, however, while their concentrations increased from 120 to 160 °C, they were then reported to substantially decrease till 240 °C; this behavior has been observed both treating AGS [90] and municipal ADSS [81]. Nevertheless, the amount of released proteins and carbohydrates from AGS was much higher than that from ADSS, probably because the AGS had a higher organic matter content (VS content) which mainly consists of proteins and carbohydrates.

### 5.3. Nutrients

HTC process waters from digestate are characterized by a relatively high nutrient content, nitrogen and phosphorous. However, nutrient content depends on HTC operative conditions and initial feedstock composition, including the presence of metals. 

#### 5.3.1. Nitrogen

Depending on the feedstock, HTC at different temperatures affects the distribution of nitrogen in HTC by-products, as a result of hydrolysis of proteins and the ammonium release. N-containing compounds are mainly present in HTC solid and liquid by-products, while N-containing gas is negligible [83]. He et al. [28] found that after HTC treatment of dewatered ADSS, N tended to release into process waters: 40% of N remained in the hydrochar while almost 60% was released into liquid phase. HTC temperature can strongly influence N distribution in HTC by-products. Yu et al. [90] reported that N distribution in the solid decreased significantly, increasing the HTC process temperature from 160 °C to 240 °C. This aspect is very important when hydrochar has to be used as an energy carrier, as the N component in fuels is transformed into NO_X_ emissions during the combustion process. Concerning N in HTC process waters, it can be present as organic nitrogen and ammonium nitrogen (NH_4_^+^-N). Organic-N is the dominant form in the water phase from lower processing temperatures (<250 °C) [91]. The effect of HTC processing temperature (160–250 °C) on the nutrient behavior in the ADSS process waters was investigated by Aragón-briceño et al. [34]. They reported the distribution of organic N and NH_4_^+^-N in process waters at different levels of process severity: while organic-N is the dominant form in process water for low processing temperatures (160 °C), the levels of nitrogen in the form of NH_4_^+^-N significantly increased in the aqueous phase as process severity increased, from 30% at 160 °C, to 40% at 220 °C, to 45% at 250 °C, respectively. Similarly, Shen et al. [84] showed that both increasing HTC temperature and treatment time would favor transformation from organic nitrogen to ammonium nitrogen. Performing HTC on ADSS, Yu et al. [81] reported that ammonium concentration increased from 320 to 490 mg N L^−1^ with HTC temperature increasing from 120 to 240 °C. However, the maximum increase in NH_4_^+^-N was observed when temperatures higher than 180 °C were applied, for which a maximum decrease in protein was also measured. He et al. [59] studied the effect of temperature from 200 to 380 °C on nitrogen content in process waters from ADSS. Total dissolved nitrogen in the process water tended to decrease with elevated temperatures, while ammonium started to increase. The same authors showed that even if a high moisture content could favor hydrolysis, increasing the moisture content of the initial digestate, the dilution effect dominates the nitrogen evolution in the process waters, thus reducing the concentration of total dissolved nitrogen and ammonia. He et al. [83] confirmed the catalysis role of CaO in promoting hydrolysis or cracking of N-containing compounds, as both NH_4_^+^-N and organic N increased substantially in process waters. Additionally, as the Ca/C molar ratio was raised from 0.05 to 0.2, NH_4_^+^-N further increased, reaching the 83% of total nitrogen in process waters, while organic N decreased. This high NH_4_^+^-N content could benefit the downstream N recovery as struvite or ammonium salts.

The HTC reaction time seems to have a secondary role. Yu et al. [81] detected the highest ammonium concentration in the process waters after only 30 min of the HTC treatment. Longer HTC process times did not increase the N concentration. Rather, an increase in treatment time may lead to the decrease in ammonium concentration in process waters, mainly due to the co-precipitation phenomena and loss of ammonia due to stripping.

Finally, nitrogen distribution also depends on the initial type of digestate treated in the HTC process. Depending on biomass treated in AD systems, different concentrations of ammonium can be reached in digestate and, thus, in HTC process waters. A very high value of total nitrogen, 18,610 mg N L^−1^, was reported by Ekpo et al. [91] for HTC process waters (250 °C, 1 h) obtained from an agro-industrial digestate, due to the co-digestion with manure. The same authors reported that the level of nitrogen in process waters in the form of NH_4_^+^-N was 55%, while that of organic nitrogen was 45%. Lower concentrations were reported for HTC process watersfrom ADSS. Interestingly, Yu et al. [90] found that nutrients in the smashed AGS tended to release more into the liquid than those in the unbroken AGS, indicating that the granule shape may help to entrap the nutrients into the hydrochar after HTC. This could suggest introducing some disintegration treatments of sludge prior to the HTC process in order to favor N release in process waters, and thus N recovery.

#### 5.3.2. Phosphorus

HTC substantially stabilizes phosphorus (P) during the processes [88], as P is mainly immobilized and retained within the hydrochar. Shi et al. [85] reported the redistribution of P and the major metals closely related to the existing forms of P in process waters and hydrochar after HTC of ADSS. Very small amounts of P, Ca, Al, Fe and Mn were detected to be released into process waters (<0.6%) while the majority of them were accumulated in the hydrochar, and the extent of this kind of accumulation increased with the increase of treatment temperature. Performing HTC on ADSS, Wang et al. [78] reported that P was mostly accumulated in the hydrochar, which agreed with the results found by Heilmann et al. [27], demonstrating that the vast majority of P forms could be recovered from hydrochar, due to precipitation of phosphate salts. On the contrary, in their study, Aragón-briceño et al. [34] reported that P was prevalently in the process waters rather than hydrochar, mainly in the form of inorganic P rather than organic P. The different fates of P during HTC, both extraction and immobilization in hydrochar, depend on HTC conditions, feedstock type and the content of inorganics, such as Al, Ca, Fe and Mg, in the feedstock [88,91]. Shi et al. [85] concluded that P speciation changes during HTC may be collectively controlled by the composition and states of metals with high affinity to phosphate and the thermochemical reactions occurring during the process.

In their recent study, Huang and Tang [88] reported speciation of P both in the untreated digestate and hydrochar. They explained that the P speciation (e.g., chemical state and physical distribution) intrinsically determines P solubility, mobility and bioavailability. Indeed, P can exist in several molecular moieties (e.g., organic phosphates, orthophosphate, phosphonate and polyphosphate) and each moiety can be present in several chemical states (e.g., to be adsorbed on other solid surfaces, complexed with cations, incorporated into mineral phases, or precipitated as P-containing solids). Raw ADSS is dominated by orthophosphate and long-chain polyphosphates [99]. Huang and Tang [88] reported that raw ADSS was characterized by:

- Al-associated P species (AlPO_4_, 40%);
- organic P (phytic acid, 20%);
- Fe/Ca-associated P species (ferrihydrite-adsorbed phosphate 13%, octacalcium phosphate, 16%);
- alumina-adsorbed phosphate (11%).

Similarly, Shi et al. [85] reported that ADSS has phosphorus mainly in the form of inorganic P (mainly in the form of apatite, Ca_5_(PO_4_)_3_), while its organic P was very low. Further, they also reported the presence of Ca (14.84%), followed by Fe (5.34%), Al (0.73%) and Mn (0.04%) in ADSS. The presence of these four metals may strongly influence the behavior of phosphorus during the HTC process.

Hydrolysis, decarboxylation and polymerization in the HTC process are responsible for the hydrolysis of polyphosphate into inorganic orthophosphate and further exposed the intracellular and organic-bound P to metals such as Ca, Fe and Al, which have a higher affinity to phosphate than metals such as Na, K and Mg, and are more abundant than metals such as Cu and Zn [99]. As a consequence, phosphates are more likely associated with Ca, Fe and Al, and can precipitate in the hydrochar as phosphate salts [85,99]. Moreover, the relative abundance and forms of these metals determine the P association stoichiometry and capacity (e.g., Fe is mostly present as hydroxide minerals and bound P as surface-adsorbed form, while Ca forms Ca-phosphate minerals in competition with its carbonate and sulfate mineral phases).

In their study on hydrochar, Huang and Tang, [88] found that Fe-associated P increased with increasing HTC treatment time, mainly in the form of ferrihydrite-adsorbed phosphate (from 13% in the raw digestate to 25–27% in the hydrochar). The abundance of total Al-associated P (AlPO_4_) decreased from 40% in the raw digestate to 7–19% in the hydrochar, while that of alumina-adsorbed P increased from 11% to 20–24%. The relative abundance of octacalcium phosphate remained unchanged [88,99]. On the contrary, Shi et al. [85] found in their study that phosphorus was mainly transformed from non-apatite inorganic P (the P fraction associated with oxides and hydroxides of Al, Fe and Mn) into apatite-P (the P fraction associated with Ca), mainly because of the high Ca content in the tested sludge and the basic condition of their experiment, which favored the dissolution of non-apatite inorganic P Equation (1) and the release of PO_4_^3−^ that could combine with Ca to form the relatively stable Ca-bound P Equation (2). This transformation increased with increasing HTC treatment temperature from 200 to 280 °C, while a sharp decrease occurred in correspondence to the organic P fraction in the hydrochar.

AlPO_4_ + 4OH^−^ ↔ (Al(OH)_4_)^−^ + PO_4_^3−^ (1)

2PO_4_^3−^ + 3Ca^+^ ↔ Ca_3_(PO_4_)_2_ (2)

The effect of different feedwater pH values on the P transformation in hydrochar during the HTC of the ADSS has been studied by Wang et al. [78]. Their results provide a theoretical basis for selecting appropriate methods and strategies for improving the performance of P recycling and reclamation from similar solid waste materials. An acidic feedwater pH promoted the transformation of apatite phosphorus to non-apatite inorganic phosphorus, and of organic P to inorganic P. On the contrary, the non-apatite inorganic phosphorus tended to be transformed into apatite phosphorus and a small part of the inorganic P was transformed to organic P when the digestate was treated in a basic environment.

Ekpo et al. [91], processing an agro-industrial digestate, showed that the extraction of total phosphorus in process waters reduces with reaction severity. During the HTC process (250 °C, 1 h), approximately 7% of the total P was extracted from digestate in the process waters. The levels of total phosphorus gradually reduced as the reaction severity increased to less than 5%. After HTC at 250 °C organic-P in process waters accounted for about 15% of the total TP concentration, while 85% was in the form of phosphate-P. Similarly, Aragón-briceño et al. [34] reported that HTC at low HTC temperatures favors the extraction of organic-P (a complex fraction of phospholipids, DNA and phosphate monoesters) in process waters, which is then broken down into inorganic P (phosphate-P). In their study, Aragón-briceño et al. [34] observed that the organic phosphorus fraction in the liquid phase, which was initially 64.4% of the total P present in the raw digestate, increases after 30 min of HTC treatment at 160 °C and 250 °C to 79.7% and 86.6%, respectively. This was accompanied by a reduction in P content in the solid fraction from 21.7% in the raw digestate to 19.4 and 11% in the hydrochar after treatment at 160 and 250 °C, respectively. Furthermore, in their study, the concentration of orthophosphate in process waters decreased between 25–32% after HTC, probably due to precipitation of some of the inorganic P with metals such as Al, Ca, Fe and Mg, forming insoluble phosphate in colloidal forms or bound to protein. 

Yu et al. [81] reported that P in HTC process waters was mainly orthophosphate, which is the reactive P for struvite precipitation. They observed an increase of both orthophosphate and ammonium from 120 to 200 °C after 30 min of HTC treatment, while a decrease in levels of both nutrients was observed for higher temperatures. The concentrations of orthophosphate and ammonium were found to drop when HTC lasted for 60 min, probably due to the precipitation of P minerals with or without ammonium, and loss of ammonia due to stripping process.

### 5.4. Heavy Metals

The HTC process influences the migration and transformation behaviors of heavy metals in solid–liquid phases of sludge. Even if the majority of heavy metals will be concentrated into the hydrochar, a certain amount can be found in the HTC process waters, representing an environmental issue [96]. Recently, in their experiments, Xu and Jiang [69] demonstrated that high HTC temperatures (240–300 °C) promoted the transition of heavy metals in hydrochar from an exchangeable state (adsorbed on the surface) to residual states. Shi et al. [86] carried out the HTC of ADSS at temperatures of 170, 200 and 280 °C. The amount of heavy metals released into process waters increased with the increase of temperature for Zn, Cd and Pb, while the higher release was obtained at 200 °C for Cr, Ni and Cu. Meanwhile, with the increase of reaction temperature, the total content of heavy metals in hydrochar increased.

Studying the HTC process waters from ADSS, He et al. [59] found that while Cu and Pb concentrations remained almost constant, the concentrations of Cr, Zn, Ni, Ca, Al and Fe decreased as the temperature increased from 200 to 380 °C. In particular, Ni was not detectable for temperatures higher than 260 °C, showing the best immobilization performance and Cr precipitation was enhanced by high temperature. During the HTC process, metals are readily transformed into dissolved ions, and metal sulfidation may take place after cooling down, for example in the form of ZnS [100]. Moreover, dissolved Ca, Al, Fe and P could interact with each other to form more stable structures [59]. Their concentrations decreased with the increase of temperature, together with P concentrations.

The dissolution of heavy metals into process waters from sewage sludge is to be coupled with the dissolution of total phosphorus [86], which could represent a chance for heavy metal adsorption, precipitation and thus immobilization in the hydrochar. 

The addition of catalysts could both improve the immobilization of heavy metals in hydrochar, and simultaneously enhance the hydrochar yield [88]. He et al. [59] studied the effect of CaO on metal concentration in process waters. Under high temperatures, the use of an appropriate amount of CaO in the HTC process has a positive effect on the immobilization of heavy metals and mineral elements for further recovery. For example, as a consequence of CaO addition, OH^−^ concentration increased, thus facilitating the dissolution of AlPO_4_ to release PO_4_^3−^, which subsequently may react with Ca_2_^+^ to precipitate as calcium phosphate (Ca_3_(PO_4_)_2_) [85] and with CaF_2_ to form fluorapatite (Ca_5_(PO_4_)_3_F) [101]. In the complex system under near-critical water conditions, CaO tends to go through solid–solid reactions with other components (e.g., silicon (Si) and aluminum (Al)) in the ash of the ADSS [102]. Thus, complex inorganic compounds, such as Ca_12_Al_14_O_33_ (mayenite), Ca(Al,Si)_2_O_4_ (yoshiokaite), CaFe_3_SiO_8_OH (ilvaite) [101], Ca_8_Al_4_(OH)_24_(CO_3_)(Cl) (H_2_O)_9.6_ (hydrocalumite), Ca_3_(Al,-Fe)_2_(Si,P)_3_O_12_ (garnet), (Ca,Na)_2_(Mg,Fe,Si,Al)_3_O_7_ (gehlenite) [103], could be formed. Nevertheless, Xu and Jiang [69] showed that the addition of FeCl_3_ or Al(OH)_3_ as catalysts had a negative impact on the immobilization of heavy metals, as it resulted in a significant increase in the exchangeable states of heavy metal in hydrochar rather than residual state.

In order to reduce the heavy metal content in process waters, Huang and Yuan [96] in their review proposed the co-processing of sewage sludge with other biomass with a lower content of heavy metals.

### 5.5. Toxic Compounds

The presence of potential toxic compounds in HTC process waters has been reported in the literature. Wirth et al. [89] measured in HTC process waters from ADSS cellulose-derived furfural, 5-hydroxymethylfurfural (HMF), phenol, cresol, catechol and resorcinol concentrations below the detection limit of 3.1 mg L^−1^.

Becker et al. [80] measured contents of furfural and 5-HMF, known as key HTC intermediates, in the process waters from anaerobically digested wheat straw. Their concentration was lower as compared to HTC process waters from other lignocellulosic biomasses, probably due to the reduction of the respective carbohydrate sources during AD of the original fresh wheat straw. However, both compounds reached their peak concentrations after HTC at 190 °C, 500 and 100 mg L^−1^, respectively, while decreasing for higher HTC temperatures. On the contrary, Berge et al. [23] did not report furfural and HMF in the HTC process waters from ADSS. It may be assumed that this was because they investigated by-products from a largely completed HTC process (250 °C, 20 h) where furfural and HMF were not detectable.

Phenols were further present in high concentrations (633–666 mg L^−1^) in the process waters from ADSS [82]. Becker et al. [80] further reported that the concentration of lignin-derived 2-methoxyphenol (major phenolic compound derived from the thermal degradation of the lignin fraction of biomasses) increases significantly in the HTC water phases of wheat straw digestate with each increase of the process temperature, having its peak of 270 mg L^−1^ at 270 °C. The authors suggest the use of 2-methoxyphenol as indicator of the concentration of potentially water-soluble compounds that tend to accumulate during HTC. 

Furthermore, 2-methylbenzofuran, known to have toxic effects on daphnia in concentrations below 10 mg L^−1^, has been further detected in HTC process waters from wheat straw digestate, having a significant increase with temperature, from concentrations below 10 mg L^−1^ at 190 and 230 °C up to 30–70 mg L^−1^ at temperature of 250–270 °C. In addition, several Maillard products, such as aldehydes, furans, pyrroles, pyrazines, pyridines and aromatic compounds, were identified in HTC process waters of sewage sludge, as a consequence of the interactions between the proteins and carbohydrates [13,35,58,104], and their presence should also be considered when HTC process is applied to anaerobically digestate.

The presence of twelve pharmaceutical compounds in sewage sludge and the removal by HTC have been studied by vom Eyser et al. [105], in order to improve the quality of hydrochar. Nevertheless, no information is available on the pharmaceuticals content in HTC process waters, and future studies are required.

## 6. Hydrothermal Carbonization Process Waters Valorization

Feedstock composition, together with HTC operative conditions (temperature, reaction time, pH, catalyst, and b/w ratio), affects the distribution of organic carbon, nutrients, heavy metals, pesticides, pharmaceutical compounds, hydrocarbons among the HTC product streams, and thus the management of the HTC final products, both hydrochar and process waters. To the best of our knowledge, there are only a few works on process waters utilization and valorization, mainly regarding its recirculation in the HTC treatment of dried biomasses. Even fewer have examined the valorization of process waters obtained from HTC of digestate.

As anticipated, a possible application, mainly applied in industrial treatment of dried biomass, is represented by the process waters recirculation in the HTC unit [77,106,107]. Typically, when treating dried biomass, water is added to the HTC system at a w/b mass ratio of 0.2–30 times the dry mass of biomass [17,19,77,108,109]. Thus, recirculation represents a practical operation that reduces the consumption of fresh water and the cost related to the process water treatment, which is one of the main operational challenges, increasing the overall efficiency of the system. In this context, the recirculation of HTC process waters also helps to reduce the produced wastewaters and the wastewaters treatment cost associated with HTC plant, as well as to recover heat and reduce tenfold the external heat consumption [110]. Recirculation, further, contributes to reduce the pressure and temperature requirements of the HTC process due to the formation of organic acid in the process waters that may catalyze the HTC reactions [77]. Hydrochar mass yields have been reported to be improved by recirculation practice [111], together with the percentage carbon and HHV in the hydrochar [112] and the dewatering properties of the hydrochar [110]. Nevertheless, this option is only useful and applicable with relatively dry feedstocks, and further treatments of the process waters are nevertheless required after a certain number of recirculation cycles.

When wet biomasses, such as raw digestate and sewage sludge, are treated with HTC, the liquid recirculation in the HTC process is usually not required or possible because of the high-water content of this waste. Other interesting options have been proposed to valorize process waters. As reported in Section 5, process waters generated during the HTC of biomasses present different concentrations of carbon, phosphorus, nitrogen, potassium, as well calcium and magnesium, making these waters suitable for agricultural applications, such as use as fertilizers [113]. Further, reaction temperatures above 120–180 °C provide a comprehensive sanitation in terms of biological hazards. Interestingly, the use of the aqueous phase from the HTC of microalgae as a nutrient culture medium for algal growth has been studied to reduce the algal cultivation costs and increase the overall sustainability of the process [114,115,116]. Different HTC process waters made from horse manure, spruce wood chips, wheat straw and biogas digestate from corn silage [95], as well as from swine manure and poultry manure [117], were investigated to evaluate the possibility of using hydrothermal carbonization (HTC) process waters as a liquid-based fertilizer for agricultural crop production, studying both germination and plant growth. However, it has also been reported that HTC process waters contain some compounds, such as phenols, organic acids and furans, which may, on the other hand, have some toxic effects on the development of plants. Recently, the toxicity of HTC process waters from sugarcane bagasse and vinasse to both the marine and terrestrial environment was evaluated by Fregolente et al. [118]. The authors showed that the terrestrial inhibition and the germination delay is species-dependent: concentrated process waters inhibited the seed germination process of maize, lettuce and tomato seeds. However, at lower concentrations, process waters delayed the germination process but did not make the germination process unfeasible. They concluded that if process waters are applied in specific quantities, they may be used as a fertilizer to provide the required nutrients for the initial growth of the plant and to promote root and shoot elongation. For the marine environment, results indicated that the HTC process waters were practically non-toxic; however, morphological changes of the marine organisms studied were observed for concentrations higher than 1000 mg TOC L^−1^.

Other few solutions have been proposed up to now in order to assess HTC process waters treatment and valorization. Treating lignocellulosic biomass, the HTC process waters were prevalently characterized by phenolic compounds and furan derivates, which may be desirable feedstocks for biodiesel and green-chemical production [119].

Recently, an exhaustive review underlined the potential of HTC process waters to be valorized through anaerobic digestion due to the relatively high residual carbon content [35]. This option has been investigated by several research groups as AD could actually contribute to energetically sustain the HTC thermal requirements, creating a positive energy balance: the produced biogas from AD of HTC process waters could supply part of the natural gas demand for an industrial-scale HTC plant, leading to an economic advantage. HTC process waters from different biomasses have been treated by AD, such as primary sewage sludge [120], brewer’s spent grain [104], orange pomace [121] agricultural residues [26], food waste [122], rice straw [123]. The methane yield of HTC process waters from different kinds of agricultural biomass, obtained from HTC at temperatures in the range of 190–260 °C and residence time of 1.25–14 h, is between 0.16–0.62 L CH_4_ gCOD^−1^ [92,104,121,124]. In their conclusion, Escala et al. [82] proposed a recovery step through AD for the remaining carbon in the process waters obtained from HTC of digestate of sewage sludge, even if lower methane yield can be obtained as compared with other biomasses [35]. Treating HTC process waters from ADSS, organic loading rates (OLRs) up to 5 gCOD L^−1^ d^−1^ have been applied, reaching methane yield of 0.18 L_CH4_ gCOD^−1^_added_ [89]. Higher OLRs, up to 18 gCOD L^−1^ d^−1^, were applied by Qiao et al. [98] treating HTC process waters from a mixture of primary and secondary sewage sludge in an upflow anerobic sludge blanket reactor, reaching methane yield of 0.26 L_CH4_ gCOD^−1^_added_. Aragón-briceño et al. [34] showed that HTC of process waters and slurry (hydrochar + process waters) from sewage sludge digestate at low HTC temperatures (160–220 °C) produced high level of biogas, 0.260–0.277 L_CH4_ gCOD^−1^_added_ and 0.235–0.276 L_CH4_ gCOD^−1^_added_, respectively. HTC process waters from digestate of microalgal biomass, characterized by a methane yield of 0.20–0.27 L_CH4_ gVS^−1^, have been recycled to the primary AD of microalgae, improving methane yield by 40% [93]. Oliveira et al. [26] reached a methane production of 0.010–0.021 L_CH4_ gFM^−1^_added_ (fresh matter) from HTC process waters of separated digested sludge and agro-industrial waste. Merzari et al. [35] concluded that HTC applied to sewage sludge at 180–200 °C with reaction time in the range of 60–90 min could be a good compromise between HTC energy consumption, hydrochar production and valorization, hydrochar dewaterability and biogas production from process waters. They suggested the management of HTC process waters from sewage sludge and from agricultural digestate inside an integrated wastewater treatment plant (WWTP)—HTC and Biogas Plant—HTC system, respectively, as one of the best options in view of actual HTC implementations.

Generally, HTC process waters from ADSS satisfy the anaerobic digestion needs in terms of nitrogen [34] and micronutrient [89] requirements. Contradictory results have been obtained regarding phosphorus requirements. While Wirth and Mumme [92] concluded that AD efficiency of HTC process waters from corn silage dropped after some weeks of AD operation due to a lack of both phosphorus ad sulphur as a consequence of precipitation phenomena with ferrous iron, Wirth et al. [89] showed that N, S and P content in HTC process waters from ADSS were in sufficient concentrations of around 200, 200 and 500 mg L^−1^, respectively, for the AD process development. This fact is mainly attributable to the presence of some metals (e.g., Al and Fe- compounds) in the initial feedstock that can favor the P precipitation. A practical operation mode is to dilute HTC process waters with digestate from the AD reactor, both to allow nutrient recycling and to reduce AD inhibition [125]. Problems related to the presence in HTC process waters of inhibitors to the AD, such as phenols, can be further solved with an appropriate AD inoculum adaption [126]. Wirth and Mumme [92] achieved in a biogas plant an anaerobic degradation of 80% of phenols contained in the HTC process waters from corn silage digestate. Furthermore, biochar addition in the AD of HTC process waters has been shown to improve both methane production in one-stage AD [66] and hydrogen and methane production in two-phase AD [127]. The performances of a continuously stirred-tank reactor (CSTR) and an anaerobic filter (AF) were compared by Wirth and Mumme [92] in treating HTC process water, showing a higher stability of the AF reactor.

Interestingly, Weide et al. [128] investigated the combined anaerobic and aerobic degradation of process waters from HTC of sewage sludge and wood in a continuous two anaerobic stages followed by one aerobic stage semi-industrial system. By applying two volumetric loadings, namely 0.44 and 0.88 g COD·L^−1^·d^−1^, in the anaerobic step, the authors obtained 254 and 296 L·kg_oDM_^−1^ of methane, respectively, as well as COD reductions of 58% and 55%. The final aerobic digestion step then led to total COD reductions of 78% and 71%, respectively. They also suggest to further reduce the COD content by ozone treatment, precipitation, and flocculation, and to investigate the possibility of co-digestion of HTC process waters with sewage sludge in wastewater treatment plants (WWTPs). The combination of AD with the HTC process has also been reported as a sustainable method of treating food waste in China [97]. 

HTC process waters, due to the relatively high residual carbon content, could be further used as an external carbon source in conventional nitrification and denitrification processes. However, this aspect should be investigated in future works, especially in relation to the inhibition of nitrifying and denitrifying bacteria.

Nowadays, HTC process waters treatment and valorization through resource recovery, mainly of nitrogen and phosphorus, is challenging [129]. In particular, phosphorus is an essential nutrient for all living organisms and for agriculture, as well as an important element in several industrial and technical uses. Almost 100% of the phosphorus used in Europe needs to be imported, and it is becoming increasingly difficult to supply the necessary amounts as the world’s mineral phosphate resources are finite. In the framework of circular economy, research is focusing on P recovery from municipal wastewaters, sludges and other wastes. Results provided by Wang et al. [78] represent a basis for selecting appropriate strategies for improving the performance of P recovery from biomasses that undergo the HTC process. Indeed, HTC has the potential for facilitating the recovery of nutrients, although its extraction is feedstock-dependent. 

HTC process waters contain high loads of ammonium [84,91], and in some case phosphate can also be found in relevant amounts [130]. Therefore, those waters can be suitable for P reclamation, through struvite (MgNH_4_PO_4_ · 6H_2_O) precipitation or calcium phosphate, when biomasses and sludges with low (multivalent) metal content are HTC-treated [81], such as sludge derived from enhanced biological phosphorus removal (EBPR), where hydrothermal phosphate release is not interfered with by high loads of metals like aluminum or iron. 

Nevertheless, conventional sewage sludge, and consequently ADSS, is usually produced by P-precipitation with the addition of metal salts like iron sulfate or sodium aluminate [49]. Therefore, phosphate tends to stay, even under acidic hydrothermal conditions, in very stable metal associations and is fixed in the hydrochar. Becker et al. [129] proposed a combination of HTC treatment of an ADSS and mechanically dewatered municipal sludge with a nutrient recycling strategy via precipitation of phosphate and nitrogen as struvite. The dewatered ADSS used in the HTC treatment was characterized by a high aluminum- and iron- salts content that compromised the phosphate release in the HTC process waters, thus, the HTC process was followed by an acid leaching step in order to remove P from hydrochar: a concentration of about 2400 mg PO_4_-P L^−1^ was reached in the process waters as compared to a concentration of about 30 mg PO_4_-P L^−1^ without the leaching step. Further, the authors used nitric acid as a catalyst in the HTC process in order to both improve the degree of carbonization and increase the amount of ammonium available in HTC process waters (up to about 4000 mg NH_4_^+^-N L^−1^) for struvite formation (a stoichiometric relation of NH_4_: PO_4_ close to 1 was achieved). After adjusting pH to 9.0 (NaOH) and the addition of a magnesium source (MgCl_2_) in a molar ratio of 1.3 Mg: PO_4_, struvite was rapidly precipitated in high purity, reaching a total recovery of phosphate from digestate of about 80%.

An integrated system of HTC and air stripping (pH 10, 55 °C, air to liquid volume ratio per minute of 5:1) was developed to effectively remove and recover nitrogen via ammonium sulphate ((NH_4_)_2_SO_4_) from dewatered ADSS [83]. In their experiment, the authors used CaO at 380 °C as a catalyst to improve hydrolysis or cracking of N-containing compounds, benefiting the downstream N recovery. The system achieved an overall N recovery rate of 62% as ammonium sulphate. Heilmann et al. [27] achieved a phosphate recovery, mainly as calcium phosphate, in yields of 80–90% from hydrochar by a combination of acid treatment (with HCl) of the hydrochar derived from HTC of animal manure, together with filtration, addition of base (NaOH) to acid extracts to achieve a pH of 9, and filtration of precipitated material.

Finally, the extraction of some organic compounds from HTC process waters could be of interest in organic syntheses and green chemistry.

## 7. Industrial Implications

Nowadays, several companies have started to industrially commercialize HTC technology, while also promoting HTC process waters valorization. In accordance with national legislations, some of them valorize the direct use of HTC process waters as fertilizer, while others propose several treatments. Ingelia, Spain, has proposed in Spain the use of HTC process waters from grass clippings and prunings as fertilizer of citrus. The same company has proposed in Italy (i) the ultrafiltration and reverse osmosis treatments of HTC process waters in order to obtain a concentrate rich in nutrients (NPK) that can be commercialized in the organic fertilizer market and a permeate that can be partially recycled to the HTC process and partially sent to the public sewage; as well as (ii) a treatment that allows to obtain a biofertilizer from the leaching of phosphorus and calcium from hydrochar mixed with other nutrients extracted from HTC process waters (nitrogen and potassium).

Other companies promote a P recovery process from both HTC process waters and hydrochar. A system able to recover calcium phosphate from hydrochar of sewage sludge has been proposed by VA GmbH-Germany. By treating the hydrochar with acid, phosphorus can be brought into the liquid phase and precipitated or crystallized almost completely as calcium phosphate, which can be used for the production of higher quality mineral fertilizers in the fertilizer industry. The same company is studying the optimization of the precipitation of a high-quality fertilizer product, magnesium ammonium phosphate (struvite). The same process is commercialized by HTCycle, Germany. Similarly, Ava-CO2, a Swiss company founded in 2009, is now commercializing a three-stage process to recover P from hydrochar (AVA cleanphos) obtained from sewage sludge. The process uses acid leaching of phosphorus from the HTC char, nanofiltration (NF) and concentration. In their plants, part of the process waters is recirculated to the HTC reactor, the rest goes through a filtration to be transferred to a WWTP.

Phosphorous recovery from the liquor from HTC of sewage sludge and the following organic fertilizer production has been industrially implemented by TerraNovaEnergy GmbH—Germany. Their technology (TerraNova® Ultra) includes an acidification step to decrease the pH of the HTC slurry below 2.0 by means of sulphuric acid, in order to have a leaching of phosphorus from the Fe- and Al- components into the liquid phase. Then, there is a slurry dewatering step in a chamber filter press that generates a filtrate to which are added calcium silicate hydrate granulates. A final dewatering step is required to separate the recovered phosphorus product, mainly composed of amorphous P-components, hydroxylapatite and struvite, which shows good plant activity. TerraNovaEnergy GmbH also operates in China. Finally, the nutrient recovery from HTC process waters derived from digestate of sludge to be used in hydroponic systems has been interestingly investigated by HBI Srl, Italy, within the project HB Ponics (HB Ponics, 2019), developing a polygenerative system through which nutrients can be recovered from the liquid phase, while producing both heat and power from the hydrochar.

## 8. Conclusions

In this work, a comprehensive analysis of the state of the art of the research on the HTC liquid phase was performed. Several works were analyzed with a particular focus on the process waters obtained from HTC of anaerobic digestate from sewage sludge and biomasses. The comparative analysis highlighted that there is a not negligible variation on the physical and chemical characteristics of process waters, which depends on both HTC process conditions and the characteristics of the raw material.

This variability can influence the valorization pathway and can represent a sort of limiting factor when applying this process at the industrial level. This work also reported a brief analysis of companies that are working on HTC, with a particular focus on their solutions regarding the valorization of the HTC liquid phase.

## Figures and Tables

**Table 1:** Digestate characteristics.

Reference	Alburquerque et al. (2012) [53]	Alburquerque et al. (2012) [53]	Alburquerque et al. (2012) [53]	Alburquerque et al. (2012) [53]	Uysal et al. (2010) [54]	^a^ Peng and Pivato, (2019) [55] ^b^ Tampio et al. (2016) [56]
Digestate	Pig Slurry and Energy-Crop Residues	Pig Slurry and Animal By-Products	Cattle Manure and Glycerin	Cattle Manure and Agro-Industrial Residues	Municipal Sewage Sludge	Organic Solid Waste
pH	7.80–7.90	7.86–8.20	5.64–7.35	7.50–7.90	7.6	7.60–8.30 ^a^
EC [dS m^−1^]	23.3–26.0	21.1–30.8	5.20–14.5	8.7–25.7	-	-
TS [g L^−1^]	28.3–43.9	19.5–29.5	17.6–72.9	17.6–90.1	25.3 ± 0.2	7.2–78.8 ^a^
TOC [g L^−1^]	8.3–14.7 ^a^	5.8–8.4	8.3–42.8	5.8–33.7	-	-
COD [g L^−1^]	3.7–4.3	1.2–3.5	8.2–27.6	1.0–5.4	25.8 ± 1.9	21.8–100.3 ^b^
BOD_5_ [g L^−1^]	4.0–6.5	2.2–6.2	10.6–52.5	1.2–5.9	0.4 ± 0.03 (as SCOD)	7.3–15.4 (as SCOD) ^b^
TN [g L^−1^]	3.4–3.6	2.9–4.9	0.6–2.3	1.4–4.0	1.0 ± 0.02	4.7–8.7 ^b^
NH_4_^+^-N [g L^−1^]	2.6–2.9	2.2–3.5	0.4 – 1.0	0.8–2.4	0.9 ± 0.01	1.7–27.5 ^a^ 1.7–4.5 ^b^
TP [g L^−1^]	1.2–1.2	0.2–0.8	0.8–1.8	0.2–0.8	0.39 ± 0.003	-
PO_4_^−^-P [g L^−1^]	-	-	-	-	0.021 ± 0.0	-
K [g L^−1^]	2.7–3.1	2.0–3.1	0.8–1.8	1.1–3.1	0.074 ± 0.005	-
Al [mg L^−1^]	-	-	-	-	91 ± 10	-
S [mg L^−1^]	367–417	219–680	48–265	113–457	-	-
Ca [mg L^−1^]	1863–1993	218–828	192–1753	1008–4026	1049 ± 57	-
Mg [mg L^−1^]	633–721	67–365	79–333	257–698	194 ± 1.5	-
Na [mg L^−1^]	666–699	696–995	66–1842	276–746	175 ± 8.2	-
Cl [mg L^−1^]	1495–1613	1598–2120	448–685	452–1418	-	-
Fe [mg L^−1^]	143–224	22–63	95–165	30–301	318 ± 32.5	-
Mn [mg L^−1^]	23–31	2.9–15.4	3.2–17.1	6.0–27.5	3.6 ± 0.1	-
Zn [mg L^−1^]	45.9–62.5	34.7–140.2	10.6–28.3	7.7–27.7	51 ± 5.4	56–300 ^a^
Cu [mg L^−1^]	7.0–8.4	4.0–15.1	1.4–13.0	2.8–10.8	4.0 ± 0.1	14–80 ^a^
B [mg L^−1^]	2.7–3.2	2.2–3.1	1.3–4.8	1.7–3.5	-	-

SCOD = soluble chemical oxygen demand. ^a^ Peng and Pivato, (2019) [55], ^b^ Tampio et al. (2016) [56].

**Table 2:** Hydrothermal carbonization (HTC) process applied to digestate.

HTC Feedstock	Laboratory Treatment Prior to HTC	Reactor Volume	HTC Conditions	Studied Products/Characteristics	Reference
ADSS	-	160 mL	250 °C, 20 h	Process waters Hydrochar	Berge et al. (2011) [23]
ADSS	-	500 mL	160, 220, 250 °C, 30 min	Process waters Hydrochar	Aragón-briceño et al. (2017) [34]
ADSS	-	200 mL	120–240 °C, 1–60 min	Process waters Hydrochar MAP precipitation	Yu et al. (2017) [81]
Dewatered ADSS (solid)	Water dilution Use of CaO additive	1000 mL	200 to 380 °C, 20 min 500 rpm	Process waters Hydrochar MAP precipitation	He et al. (2015) [59]
Dewatered ADSS (solid)	Water dilution in order to obtain a TS content of 20% Use of citric acid as catalyst	25.0 L	205 °C, 7 h pH regulation by acetic acid and sodium hydroxide	Process waters Hydrochar Dewaterability	Escala et al. (2013) [82]
Dewatered ADSS (solid)	Pre-dried at 105 °C for 12 h Water dilution	1000 mL	200 to 380 °C, 20 min 500 rpm	Process waters Hydrochar Ammonia stripping	He et al. (2015) [83]
Dewatered ADSS (solid)	-	200 mL	160, 200, 240 °C, 4, 8, 12 h	Process waters Hydrochar N distribution	Shen et al. (2018) [84]
Dewatered ADSS (solid)	Water dilution Addition of Cd	200 mL	200, 280 °C, 1 h	Process waters Hydrochar P distibution	Shi et al. (2014) [85]
Dewatered ADSS (solid)	Water dilution Addition of Cr, Ni, Cu, Zn, Cd, Pb	200 mL	170, 200. 280 °C, 1 h	Process waters Hydrochar P and HM distribution	Shi et al. (2013) [86]
Dewatered ADSS (solid)	-	125 mL	200 °C, 4, 6, 8, 10, 12 h	Hydrochar	He et al. (2013) [28]
ADSS	Water dilution	1000 mL	180–200 °C, 30 min 200 rpm	Hydrochar Dewaterability	Kim et al. (2014) [32]
ADSS	Water dilution	200 mL	200 °C, 24 h	Hydrochar	Alatalo et al. (2013) [87]
Dewatered ADSS (solid)	Pre-dried at 105 °C for 24 h Triturated	500 mL	200, 230, 260 °C, 2 h	Hydrochar P evolution	Wang et al. (2017) [78]
Dewatered ADSS (solid)	Water dilution	20 mL	225°C, 4–16 h	Hydrochar P distribution	Huang and Tang (2016) [88]
ADSS		3.0 m^3^	200 °C, 6 h pH regulation by citric acid	Process waters HTC + AD	Wirth et al. (2015) [89]
AGS	-	200 mL	160, 200, 240 °C, 1 h	Process waters Hydrochar AD+HTC	Yu et al. (2018) [90]
ADSS		3.0 m^3^	200 °C, 6 h pH regulation by citric acid	Condensate	Wirth and Reza (2016) [70]
Anaerobically digested wheat straw (thermophilic digestion)	Pre-dried at 60 °C for 72 h Water dilution in order to obtain a carbon concentration of 26.6 g L^−1^	1000 mL	190, 210, 230, 250 °C, 1, 2.5, 4 h	Hydrochar	Funke et al. (2013) [33]
Anaerobically digested maize silage (thermophilic digestion)	Water dilution in order to obtain a carbon concentration of 42.3 g L^−1^	1000 mL	190, 230, 270 °C, 2, 6, 10 h 90 rpm pH regulation by citric acid (pH 3, 5, 7)	Hydrochar	Mumme et al. (2011) [10]
Anaerobically digested wheat straw (thermophilic digestion)	Water dilution in order to obtain a TS content of 10%	1000 mL	230 °C, 6 h 90 rev/min	Hydrochar	Mumme et al. (2014) [66]
Dried anaerobically digested cow manure and maize (mass ratio of 4:3 as feedstock) and zeolite	Pre-dried at 105 °C for 24 h Cut to particle size below 1 mm Water dilution	1000 mL	190, 230, 270 °C, 2 h 90 rev/min Addition of zeolite	Hydrochar–zeolite composite	Mumme et al. (2015) [79]
Anaerobically digested agro-industrial biomass	Pre-dried at 60 °C for 48 h	75 mL	250 °C, 1 h	Process waters Hydrochar	Ekpo et al. (2016) [91]
Anaerobically digested wheat straw (thermophilic digestion)	Pre-dried at 60 °C for 48 h Water dilution in order to obtain a carbon concentration of 26.7 g L^−1^	125 mL	190, 230, 250, 270 °C, 6.0 h	Process waters	Becker et al. (2014) [80]
Anaerobically digested corn silage	-	Full-scale plant located at Karlsruhe, Germany	220 °C, 6.0 h	Process waters Valorization process waters througth AD	Wirth and Mumme (2014) [92]
Dewatered anaerobically digested algal biomass	Water dilution	300 mL	200 °C, 1 h	Process waters HTC + AD system	Nuchdang et al. (2018) [93]
Dewatered anaerobically digested agro-industrial biomass		25.0 L	180 °C, 4 h	Process waters Hydrochar	Oliveira et al. (2013) [26]
Dewatered anaerobically digested municipal solid waste	-	100 mL	200, 250, 300 °C 0.5, 2 h 90 rpm	Process waters, Hydrochar	Reza et al. (2016) [94]
Anaerobically digested corn silage		Full-scale plant, Germany	180 °C, 8–10 h	Process water Hydrochar Toxicity	Bargmann et al. (2013) [95]

CaO = Calcium oxide; TS = Total Solid; AD = Anaerobic Digestion; AGS = Anaerobic Granular Sludge; ADSS = Anaerobic Digested Sewage Sludge.

**Table 3:** Hydrothermal carbonization (HTC) process waters compositions.

References	Raw Material and HTC Conditions	Yield	pH	TOC	Soluble COD	VFAs	Acetic Acid	TS	Total N	NH_4_^+^-N	Total Soluble P	Ortho-P	Total K	Phenols	Others	C	H	N	S	O
			-	mgC L^−1^	mg L^−1^	mgCOD L^−1^	mg L^−1^	%	mgN L^−1^	mgN L^−1^	mgP L^−1^	mgP L^−1^	mgK L^−1^			%	%	%	%	%
Berge et al. (2011) [23]	ADSS																			
	HTC 250 °C, 20 h	-	8.0	4000	10000									YES ^1^	YES ^1^					
Aragón-briceño et al. (2017) [34]	ADSS		7.8	461.6	1843	4.8		4.5	1493	1344	91.3	80.1				30.5	4.4	10.2	0.7	54.1
	HTC 160 °C, 30 min		9.1	4686	12,642	191.1			2066	1258	94.0	53.9				45.8	6.8	11.1	1.9	34.5
	HTC 220 °C, 30 min		7.1	4584	12,992	406.0			2191	1704	72.6	59.8				49.2	6.3	12.3	2.4	29.8
	HTC 250 °C, 30 min		8.1	4879	12,164	715.7			2354	1685	103.8	56.8				68.0	6.6	6.6	1.8	10.9
He et al. (2015) [59]	Dewatered ADSS							17.5												
	HTC 200 °C, 20 min	87 (%vol)	8.6	24,070	63,900				12,000	4020	*246*				YES ^1^					
	HTC 280 °C, 20 min	98 (%vol)	8.4	16,000	40,000				10,100	6400	*191*									
	HTC 380 °C, 20 min	95(%vol)	8.1	12,510	30,400				10,000	7980	*89*									
	HTC 380 °C, 20min, CaO		10.0	18,630	55,800				12,000	8700	*21*									
Escala et al. (2013) [82]	Dewatered ADSS		6.9–7.4					23.9												
	HTC 205°C, 7h, Ca		7.0		53,000				2590	2047	14.3	11.5		666						
	HTC 205°C, 7h		6.9		40,600				2710	2153	17.8	4.8		633						
Shi et al. (2014) [85]	Dewatered ADSS		6.4					15	3150		3900									
	HTC 200 °C, 1h										0.2%^+^									
	HTC 280 °C, 1h										0.6%^+^									
Yu et al. (2017) [81]	ADSS		6.3	1800				1.8		300		370								
	HTC 160 °C, 30 min		6.0	4000						400		480								
	HTC 200 °C, 30 min		5.7	5000						450		570								
	HTC 240 °C, 30 min		5.5	6000						490		400								
Shi et al. (2013) [86]	Dewatered ADSS		6.4					14.5												
	HTC 170 °C, 1 h		7.6						2357		12.5									
	HTC 200 °C, 1h		8.5						2586		15.8									
	HTC 280 °C, 1 h		9.2						3566		30.4									
Wirth et al. (2015) [89]	ADSS																			
	HTC 200 °C, 6 h, pH regulation		4.7	13,400	34300		2060	3.4	2800	1000					YES ^2^					
Yu et al., (2018) [90]	AGS		6.8	1118		100	0	9.5												
	HTC 160 °C, 1 h		6.0	15,611		454	300													
	HTC 200 °C, 1 h		5.8			1100	900													
	HTC 240 °C, 1 h		5.6			2557	2000													
Ekpo et al. (2016) [91]	Agro-industrial digestate																			
	HTC 250 °C, 1 h	30 (wt%)	7.7	62,350					18,610	10,235	840		2340							
Becker et al. (2014) [80]	Wheat straw digestate																			
	HTC 190 °C, 6.0 h		4.0	5800			1000							YES ^3^	YES ^3^					
	HTC 230 °C, 6.0 h		4.0	9000			1000													
	HTC 250 °C, 6.0 h		4.0	7800			1250													
	HTC 270 °C, 6.0 h		4.0	9500			1200													
Wirth and Mumme (2014) [92]	Corn silage digestate																			
	HTC 220 °C, 6.0 h		3.88	15,660	41,350		5260	2.8	685	229	197			*290*						
Nuchdang et al. (2018) [93]	Microalgae digestate				1926			0.9												
	HTC 200 °C, 1.0 h				8204			0.9												
Reza et al. (2016) [94]	MSW digestate		8.1					23												
	HTC 200 °C, 30 min		8.2																	
	HTC 200 °C, 2.0 h		8.3																	
Bargmann et al. (2013) [95]	Corn silage digestate																			
	HTC 180 °C, 9.0 h		5.7							83.3	20.3		328							

^1^ 1-Methyl-4-[nitromethyl]-4-piperidinol; 1-Methyldodecylamine; 1-Phenethyl-piperidin-4-ol; 1-Propanol, 2-amino-; 2,5-Pyrrolidinedione, 1-ethyl-; 2,5-Pyrrolidinedione, 1-methyl-; 2-Butanamine, (S)-; 2-Cyclopenten-1-one, 2,3-dimethyl-; 2-Cyclopenten-1-one, 2-methyl-; 2-Heptanamine, 5-methyl-; 2-Propanamine; 3-Aminopyridine; 3-Buten-2-one, 3-methyl-, dimethylhydrazone; 3-Cyclohexene-1-carboxaldehyde, 4-methyl-; 4-Fluorohistamine; Acetic acid; Benzoic acid, 2,4-dihydroxy-, (3-diethylamino-1methyl)propyl ester; Dimethylamine; dl-Alanine; Formic acid phenyl ester; Hydrogen chloride; Methylpent-4-enylamine; Phenethylamine, p-methoxy-.alpha.-methyl-, (.+/-.)-; Phenol; Phenol, 4-methyl-; Pyrazole, 1-methyl-4-nitro-; Tetrahydro-4H-pyran-4-ol. ^2^ Furfural, 5-HMF, phenol, cresol, catechol, and resorcinol. ^3^ Furfural (50–500 mg L^−1^), 5-HMF (0–100 mg L^−1^), phenolic compounds (2-methoxyphenol) (10–300 mg L^−1^), 2-Methylbenzofuran (2–70 mg L^−1^) ^+^, expressed as redistribution of phosphorus in process waters (%). TOC = Total Organic Carbon COD = Chemical Oxygen Demand VFA = Volatile Fatty Acids NH_4_^+^-N = ammonium nitrogen.

**Table 4:** Compounds identified in the HTC process water from HTC of anaerobically digested sewage sludge (ADSS) and their main possible application. Adapted from Berge et al. [23].

Compound	Application
1-Methyl-4-[nitromethyl]-4-piperidinol	Production of antitumor agents and products involved in the treatment of cardiovascular diseases
1-Methyldodecylamine	Preparation of N,N,N,N,N,N- trimethyldodecylammonium bromide
1-Phenethyl-piperidin-4-ol	-
1-Propanol, 2-amino-	Organic syntheses (e.g, Schiff base ligands)
2,5-Pyrrolidinedione, 1-ethyl-	Organic syntheses
2,5-Pyrrolidinedione, 1-methyl-	Organic syntheses, as well as in some industrial silver-plating processes
2-Butanamine, (S)	Production of some pesticides
2-Cyclopenten-1-one, 2,3-dimethyl-	-
2-Cyclopenten-1-one, 2-methyl-	-
2-Heptanamine, 5-methyl-	-
2-Propanamine	Production of some herbicides and pesticides including atrazine, bentazon, glyphosate; agent for plastics; intermediate in organic synthesis of coating materials, pesticides, plastics, rubber chemicals, pharmaceuticals and others; additive in the petroleum industry
3-Aminopyridine	Synthesis of organic ligand 3-pyridylnicotinamide.
3-Buten-2-one, 3-methyl-, dimethylhydrazone	-
3-Cyclohexene-1-carboxaldehyde, 4-methyl-	-
4-Fluorohistamine	Organic syntheses
Acetic acid	Production of cellulose acetate for photographic film, polyvinyl acetate for wood glue, and synthetic fibers and fabrics; descaling agent, used in the food industry, in biochemistry
Benzoic acid, 2,4-dihydroxy-, (3-diethylamino-1- methyl)propyl ester	-
Dimethylamine	Dehairing agent in tanning, in dyes, in rubber accelerators, in soaps and cleaning compounds; agricultural fungicide
dl-Alanine	Food and pharmaceutical industry; plating chemicals and animal feed
Formic acid phenyl ester	Used for palladium-catalyzed carbonylation of aryl, alkenyl and allyl halides; used as a reagent for the formulation of amines
Hydrogen chloride	Used in cleaning, pickling, electroplating metals, tanning leather, and refining and as an agent for producing a wide variety of products
Methylpent-4-enylamine	-
Phenethylamine, p-methoxy-.alpha.-methyl-, (.+/-.)-	-
Phenol	Precursor to many materials and useful compounds; used to synthesize plastics and related materials; production of polycarbonates, epoxies, Bakelite, nylon, detergents, herbicides such as phenoxy herbicides, and numerous pharmaceutical drugs.
Phenol, 4-methyl-	Production of antioxidants, e.g., butylated hydroxytoluene
Pyrazole, 1-methyl-4-nitro-	-
Tetrahydro-4H-pyran-4-ol	-
